# Assessment of the Population's Willingness To Consider Elderly Daycare Centres In Saudi Arabia

**DOI:** 10.7759/cureus.78778

**Published:** 2025-02-09

**Authors:** Aghadeer A Kayal, Khadijah K Angawi, Rasha R Alsaigh, Hafiz T.A. Khan

**Affiliations:** 1 Department of Health Information Management, Prince Mohammed bin Abdulaziz Hospital, Ministry of National Guard, Health Affairs, Al-Madinah al-Munawarah, SAU; 2 Department of Health Services and Hospital Administration, King Abdulaziz University, Jeddah, SAU; 3 Maternity and Child Health, King Abdulaziz University Faculty of Nursing, Jeddah, SAU; 4 Public Health & Statistics, College of Nursing, Midwifery and Healthcare University of West London, Brentford, GBR

**Keywords:** ageing population, daycare, elderly, saudi arabia, willingness

## Abstract

Background

Population ageing is a significant global challenge. Healthcare systems of all types are under great strain because of the health needs of the elderly. In this regard, one of the solutions is the utilisation of daycare facilities for the elderly. Current information shows that the topic of daycare facilities in Saudi Arabia does not receive sufficient attention. Therefore, the objective of this study was to assess the population’s willingness to consider elderly daycare for their older relatives in Saudi Arabia.

Methods

The research employs a cross-sectional study design in which questionnaires were randomly distributed to Saudi citizens aged between 18 and 59 years. A total of 730 participants were included. IBM SPSS Statistics for Windows, version 28.0. (IBM Corp., Armonk, NY), Kendall’s Tau correlation and ordinal logistic regression analysis were used to identify important factors associated with public willingness to consider their relatives in daycare centres.

Results

Around three-fifths of the Saudi population are willing to enrol the elderly in daycare institutions. People aged 45-51 years were three times more willing to consider elderly daycare institutions for their older relatives (aOR: 3.85, 95% CI: 1.72-8.33) as compared to younger aged 18-26 years. Factors that are associated with higher willingness include “Seniors play an important role in our society" (aOR: 1.67, 95% CI: 1.30-2.13) and “The elders are wise and knowledgeable about the traditions of their community” (adjusted odds ratio {aOR}: 1.49, 95% CI: 1.19-1.89). The factors associated with higher willingness are awareness regarding the existence of elderly daycare institutions in Saudi Arabia (aOR: 1.37, 95% CI: 1.11 to 1.67). Other important factors associated with a higher willingness to consider daycare are the nature of the occupation, financial capacity, being strong enough physically to handle the elderly, and understanding the elderly.

Conclusion

It has been suggested that many Saudis are fully aware of the concept of elderly daycare centres and willing to let their older relatives join them. Therefore, spreading awareness and introducing daycare centres to more people can make a difference in our community to provide the care our elderly deserve.

## Introduction

The shift of a country's population towards higher ages, known as “population ageing,” is a phenomenon experienced by every country in the world regardless of its income level. In all affluent nations, the population growth among older adults is expanding rather fast and consistently [1]. It is expected that by the year 2030, one in six people will be 60 years old or older, and the estimated world’s elderly population will be 1.6 billion [2]. Life expectancy has increased many concerns because longer life expectancies are accompanied by years of poor health and impairment, necessitating prompt medical attention [3]. As reported by Prasad et al., over 80% of older adults suffer from at least one chronic condition, such as cardiovascular disease, stroke, diabetes, dementia, or arthritis, and 50% have at least two [4]. This high prevalence of chronic conditions among the elderly highlights the need for medical treatment and care management, which puts a burden on the healthcare system. The increased proportion of older adults also poses questions about their capacity to provide adequate care. Therefore, to ensure the long-term stability of national healthcare systems, nearly every country on earth would need to significantly increase both the number of physicians and nurses as well as the resources available to provide the needed care for older adults [5]. According to the Saudi General Authority of Statistics (2021), Saudi Arabia currently has one million and three thousand senior citizens or 5% of the country's total population, and that number is predicted to rise to 25% by the year 2050 [5,6].

Since the country would otherwise experience enormous and, to a great degree, crippling demand on its current healthcare system, actions aimed at modifying the current practices are more or less imperative today. Among the most robust solutions, in this case, is the usage of daycare facilities, which can both provide advanced care to patients and, at the same time, prevent overload due to decreasing the contact period between the doctors and their clients in hospital settings [7].

One of the main roles of elderly daycare facilities is to create an opportunity for social interaction, as during the day the seniors get to spend time with other seniors while receiving care, which provides them with an opportunity to create new friendships and build peer support. On the other hand, the growing preference for elderly daycare centres is a result of the realisation that carers do not provide everything that the seniors require while they are at home. Most elderly people suffer from chronic conditions that include arthritis, heart disease, cancer, or diabetes, which require close follow-up regularly to reduce the risk of complications or infections [8]. However, how willing are Saudis to consider these elderly daycare centres for their older relatives is still a question to be asked, especially due to embedded local culture and norms that may place an assumed responsibility in providing care for their older members [9].

A relatively clear research gap arises in understanding the positive long-term impacts of using daycare facilities. Current information shows that the topic of elderly daycare facilities in Saudi Arabia does not receive sufficient attention [10]. Nonetheless, the willingness of the Saudis to consider elderly daycare centres for their older relatives is also scarce. While countries such as Japan and China make some investments in relevant research, the existing knowledge in the Arab states, for example, is far from sufficient [11]. Primarily, daycare is a setting for some research but not a full-scale review subject. In this light, the presented study closes the outlined research gap completely. The survey's main objective is to study the public's willingness to consider elderly daycare centres for their older relatives in Saudi Arabia.

## Materials and methods

Research design

A descriptive cross-sectional research design was used in an attempt to assess the willingness of the Saudi population to consider elderly daycare centres for their older relatives.

Study limits/scope

This study is limited to assessing the public's willingness to enrol their older relatives in elderly daycare institutions in Saudi Arabia. The study focused on Saudi citizens, male and female individuals, ages 18-59 years old. Participants who are 60 years old and above were excluded.

Data collection tool

The primary data source for this study was a closed-ended questionnaire using a five-level Likert scale (Appendix) that was distributed through social media platforms adopted from Chepkwony and Kiptiony [12]. The questionnaire's validity and reliability were examined using Cronbach's alpha test, and the result was considered reliable (0.862). The questionnaire consisted of five parts.

Part A: Demographic Data

This section includes eight questions: gender, marital status, age, source of income, monthly income range, size of household, education, and to what extent the participant is willing to consider elderly daycare for their older relatives. 

PART B: Public Perception of Older People

This section includes eight questions regarding the population's perception of the elderly in Saudi Arabia; for example, if older people play important roles in our society or if older people are leading solitary lives in many families.

PART C: Perceived Institutional Efficacy on Care Provision for Older People

This section includes nine questions focusing on the population's perception of the efficiency and effectiveness of elderly daycare institutions on the elderly's well-being; for example, the well-trained staff in elderly daycare institutions to take care of the elderly and the available facilities that the elderly can use for physical activities.

PART D: Perceived Older People’s Efficacy on Care Provision for Themselves

This section includes four questions discussing the elderly’s ability to take care of themselves, for example, elderly physical weakness and health issues.

PART E: Perceived Efficacy of Members of the Public (Caregivers) to Take Care of Their Older Relatives

This section includes 10 questions related to the ability of caregivers to care for their older relatives; for instance, elderly psychological problems that require regular presence and the time needed to provide care for the elderly.

Data collection method

The questionnaire was distributed electronically through all social media platforms (WhatsApp, Facebook, and Instagram) to reach as many people as possible and was answered by 730 participants.

Sampling

Purposive sampling techniques were applied for the study. Purposive sampling, also known as non-probability sampling, relies on the researcher’s judgement to choose the participants and is applied when the researcher requires all participants to fit specific characteristics and was used in this study. The inclusion criteria are the characteristics that the prospective participant must have to be included in the study. In contrast, exclusion criteria are defined as the characteristics that, if the prospective participant is presented with, they will be excluded from the study. The inclusion criteria for this study include all male and female Saudi citizens and participants between the ages of 18 and 59 years old. The exclusion criteria for this study include participants who are 60 years old and above.

Sample size and selection of sample 

Sample size calculator from https://www.calculator.net/sample-size-calculator.html was used to select the size of the sample, which was estimated to be (n = 666) with a 5% margin of error and a 99% confidence level, and a total of 730 replies were received between May 2022 and June 2022.

Statistical analysis

IBM SPSS Statistics for Windows, version 28.0. (IBM Corp., Armonk, NY), Kendall’s Tau correlation and ordinal logistic regression analysis were used to analyze the collected data. Kendall's Tau correlation was done between the willingness to consider elderly daycare for the elderly and different questionnaire items. Ordinal logistic regression was done to study the impact of independent variables on the willingness to enrol older relatives in elderly daycare institutions.

Kendall’s Tau correlation was used to study the association and strength between independent variables and the degree of willingness to enrol older relatives in elderly daycare institutions (dependent variable). Kendall’s Tau analysis shows two different results, a correlation coefficient (called “Tau”) that ranges from -1 to 1 and a p-value. The absence of a relationship between the variables is indicated by a negative Tau value or when one variable increases the other will decrease. On the other hand, a positive Tau value indicates that both variables are related and when one increases the other will increase as well.

Ordinal logistic regression was done to properly assess which of the independent variables has a statistically significant impact on the degree of willingness to enrol older relatives in elderly daycare institutions (dependent variable).

Ethical consideration

In this study, ethical approval was granted by the Research and Studies Department in the Ministry of Health, Jeddah on March 16, 2022. The ethical approval number is A0132.

## Results

A summary of the demographic traits of the respondents is provided in Table 1. Most of them were female individuals (411 {56.3%}) and 466 (63.8%) were married. Among the respondents, those with high school degrees constituted 275 (37.7%) individuals.

**Table 1:** Demographic characteristics of participants.

	N	%
Gender		
Male	319	43.7
Female	411	56.3
Social status		
Single	168	23.0
Married	466	63.8
Divorced	39	5.3
Widowed	57	7.8
Age		
18-26 years	153	21.0
27-32 years	118	16.2
33-38 years	196	26.8
39-44 years	100	13.7
45-51 years	55	7.5
52-59 years	108	14.8
Source of income		
Formal employment	165	22.6
Casual employment	305	41.8
Business	57	7.8
Others	203	27.8
Monthly income range		
Below 5000	153	21.0
5000-10,000	227	31.1
11000-15,000	182	24.9
16000-20000	113	15.5
21000-25000	22	3.0
26000-30000	13	1.8
31000 and above	20	2.7
The size of your household		
From 2-5	312	42.7
From 6-9	291	39.9
From 10-12	113	15.5
From 13-15	9	1.2
More than 15	5	0.7
Education level		
No formal education	1	0.1
Primary	2	0.3
Secondary	17	2.2
High school	275	37.7
College	132	18.1
University	244	33.3
Masters or PhD	59	8.1

Public willingness to consider elderly daycare for older relatives

Based on the responses, most participants recognize, know, or understand what daycare centres for the elderly do. In Table 2 more than three-quarters are willing or very willing to consider sending older relatives to daycare. However, although they may be knowledgeable about daycare centres, some may still avoid them due to the guilty feeling they may have to leave their older relatives in another person's care.

**Table 2:** Public willingness to consider elderly daycare for their older relative.

	N	%
Not willing	34	4.7
Least willing	52	7.1
Somehow willing	151	20.7
Willing	228	31.2
Very willing	265	36.3

Table 3 displays the questionnaire results, with each section assessing a different aspect of the topic to ensure a coherent and comprehensive assessment of public perception and efficacy related to the care of older individuals. The first section (public perception of older people) focused on measuring how the public perceived the roles and characteristics of older people in society, while the second section (perceived institutional efficacy on care provision for older people) examined the perceived effectiveness of institutions in providing care for older people. Furthermore, the other two sections (perceived older people’s efficacy on care provision for themselves and perceived efficacy of members of the public (caregivers) to take care of their older relatives) explored the perceptions of older individuals ability to care for themselves in addition to the confidence and capabilities of family members or caregivers in providing care for their older relatives.

**Table 3:** Public perception, perceived institutional efficacy and perceived efficacy about care provision to older people.

	Strongly disagree	Disagree	Somehow agree	Agree	Strongly agree	Mean ±SD	Cronbach’s alpha
	N(%)	N(%)	N(%)	N(%)	N(%)		
Public perception of older people							
Older people play important roles in our society	13 (1.8)	38 (5.2)	26 (3.6)	293 (40.1)	360 (49.3)	4.30±0.90	0.805
Older people are wise and knowledgeable about the traditions of their society	12 (1.6)	45 (6.1)	26 (3.6)	270 (37)	377 (51.6)	4.31±0.92	
Older people are less tolerated and received to changes in society	25 (3.4)	72 (9.9)	71 (9.7)	302 (41.4)	260 (35.6)	3.96±1.08	
Older people are being neglected by their dependents in many families	31 (4.2)	133 (18.2)	106 (14.5)	281 (38.5)	179 (24.5)	3.61±1.16	
Older people are leading solitary lives in many families	26 (3.6)	135 (18.5)	79 (10.8)	322 (44.1)	168 (23)	3.65±1.13	
There are many cases of elder abuse in society	35 (4.8)	161 (22.1)	121 (16.6)	273 (37.4)	140 (19.2)	3.44±1.17	
Older people are respected and valued in society	21 (2.9)	65 (8.9)	48 (6.6)	324 (44.4)	272 (37.3)	4.04±1.03	
Older people are warm and welcoming to the younger generation	10 (1.4)	98 (13.4)	103 (14.1)	349 (47.8)	170 (23.3)	3.78±0.99	
Perceived institutional efficacy on care provision for older people							
I am very much aware of the existence of elderly daycare institutions in Saudi Arabia	26 (3.6)	100 (13.7)	144 (19.7)	355 (48.6)	105 (14.4)	3.57±1.01	0.891
Elderly daycare institutions have good accommodation facilities to take care of my older relatives	30 (4.1)	102 (14.0)	178 (24.4)	292 (40.0)	128 (17.5)	3.53±1.06	
Elderly daycare institutions have enough facilities that my older relative can use for physical exercises	37 (5.1)	159 (21.8)	183 (25.1)	234 (32.1)	117 (16.0)	3.32±1.13	
Elderly daycare institutions for older people are operating according to government regulations	27 (3.7)	119 (16.3)	166 (22.7)	300 (41.1)	118 (16.2)	3.50±1.06	
Elderly daycare institutions have sufficient and acceptable programs and activities that my older relative can use for social bonding	42 (5.8)	136 (18.6)	203 (27.8)	235 (32.2)	114 (15.6)	3.33±1.12	
I have confidence in the individuals/organizations that have established elderly daycare institutions for older people	31 (4.2)	106 (14.5)	180 (24.7)	293 (40.1)	120 (16.4)	3.50±1.06	
Elderly daycare institutions have well-trained personnel that can take care of my older relatives	26 (3.6)	103 (14.1)	193 (26.4)	291 (39.9)	117 (16.0)	3.51±1.03	
Staff of elderly daycare institutions have positive attitudes toward older people in society	20 (2.7)	106 (14.5)	191 (26.2)	303 (41.5)	110 (15.1)	3.52±1.00	
Elderly daycare institutions have adequate staff to respond to the needs of my older	29 (4.0)	131 (17.9)	203 (27.8)	262 (35.9)	105 (14.4)	3.39±1.06	
Perceived older people’s efficacy on care provision for themselves							
My older relative is physically weak and cannot fend for him/herself	14 (1.9)	111 (15.2)	66 (9.0)	397 (54.5)	142 (19.5)	3.74±1.00	0.743
My older relative suffers from serious health problems that require regular medical checkups	9 (1.2)	90 (12.3)	40 (5.5)	347 (47.%)	244 (33.4)	4.00±1.00	
My older relative is economically unstable and cannot meet the cost of his/her medical needs (financial capacity)	26 (3.6)	126 (3.6)	68 (9.3)	307 (42.1)	203 (27.8)	3.73±1.15	
My older relative does not have enough friends to give him/her company (psychosocial status)	22 (3.0)	170 (23.3)	83 (11.4)	300 (41.1)	155 (21.2)	3.54±1.15	
Perceived efficacy of members of the public (caregivers) to take care of their older relatives							
My relationship with my older relative was very good prior to his/her old age	41 (5.6)	144 (19.7)	80 (11.0)	336 (46.0)	129 (17.7)	3.50±1.16	0.807
My current relationship with older relatives is very good and cordial	23 (3.2)	69 (9.5)	36 (4.9)	343 (47.0)	259 (35.5)	4.02±1.03	
The nature of my occupation permits me to dedicate enough time to take care of my older relative	45 (6.2)	156 (21.4)	105 (14.4)	282 (38.6)	142 (19.5)	3.44±1.20	
I have the financial capacity to take care of my older relative given his/her level of dependency	59 (8.1)	183 (25.1)	132 (18.1)	248 (34.0)	142 (19.5)	3.22±1.21	
I have adequate financial resources to cater for the medical needs of my older relative	53 (7.3)	192 (26.3)	153 (21.0)	249 (34.1)	83 (11.4)	3.16±1.15	
Am strong enough psychologically to take care of my older relative	36 (4.9)	128 (17.5)	111 (15.2)	314 (43.0)	141 (19.3)	3.54±1.13	
I have enough free time that allows me to take care of my older relative without major assistance from other people	42 (5.8)	205 (28.1)	130 (17.8)	267 (36.6)	86 (11.8)	3.21±1.14	
I understand my older relative very well, so it is easy for me to take care of him/her	22 (3.0)	146 (20.0)	105 (14.4)	330 (45.2)	127 (17.4)	3.54±1.09	
My older relative appreciates every help or support that I offer him/her	26 (3.6)	120 (16.4)	89 (12.2)	353 (48.4)	142 (19.5)	3.64±1.08	
I have the capacity to take care of my older given his/her mental/psychological state	24 (3.3)	118 (16.2)	112 (15.3)	356 (48.8)	120 (16.4)	3.59±1.05	
My older relative suffers from psychological problems that require my regular presence	22 (3.0)	169 (23.2)	139 (19.0)	300 (41.1)	100 (13.7)	3.39±1.08	

Inferential statistics

Kendall’s Tau Correlation

In Table 4, a moderate positive correlation was found between perceiving an elder’s important role in society and willingness to consider elderly daycare for older relatives. The greater the understanding of senior citizens' significant contributions to society the higher the willingness of uptake of elderly daycare services for older people (correlation coefficient = 0.338, p-value <0.001). Furthermore, a moderate positive correlation was also found between respecting the elderly and willingness to consider elderly daycare for older relatives; the more you respect the elderly, the higher the uptake of elderly daycare services for older people (correlation coefficient = 0.239, p-value <0.001). All other questions showed a weaker positive correlation with the outcome “willingness to consider elderly daycare for older relatives.” The analysis showed a weak positive correlation with the outcome “willingness to consider elderly daycare for your older relative.”

**Table 4:** Association between independent variables and the degree of willingness to enroll older relatives in elderly daycare institutions.

Independent variables	Willingness to consider elderly daycare for your older relative	
	Correlation coefficient	P-value
Public perception of older people		
Older people play important roles in our society	0.338	<0.001
Older people are wise and knowledgeable about the traditions of their society	0.334	<0.001
Older people are less tolerated and received to changes in society	0.192	<0.001
Older people are being neglected by their dependents in many families	0.137	<0.001
Older people are leading solitary lives in many families	0.145	<0.001
There are many cases of elder abuse in society	0.134	<0.001
Older people are respected and valued in society	0.239	<0.001
Older people are warm and welcoming to the younger generation	0.219	<0.001
Perceived institutional efficacy on care provision for older people		
I am very much aware of the existence of elderly daycare institutions in Saudi Arabia	0.148	<0.001
Elderly daycare institutions have good accommodation facilities to take care of my older relatives	0.077	0.013
Elderly daycare institutions have enough facilities that my older relative can use for physical exercises	0.101	0.001
Elderly daycare institutions for older people are operating according to government regulations	0.125	<0.001
Elderly daycare institutions have sufficient and acceptable programs and activities that my older relative can use for social bonding	0.100	0.001
I have confidence in the individuals/organizations that have established elderly daycare institutions for older people	0.135	<0.001
Elderly daycare institutions have well-trained personnel that can take care of my older relatives	0.114	<0.001
Staff of elderly daycare institutions have positive attitudes toward older people in society	0.138	<0.001
Elderly daycare institutions have adequate staff to respond to the needs of my older	0.101	0.001
Perceived older people’s efficacy on care provision for themselves		
My older relative is physically weak and cannot fend for him/herself	0.068	0.031
My older relative suffers from serious health problems that require regular medical checkups	0.076	0.017
My older relative is economically unstable and cannot meet the cost of his/her medical needs (financial capacity)	0.023	0.458
My older relative does not have enough friends to give him/her company (psychosocial status)	0.041	0.186
Perceived efficacy of members of the public (caregivers) to take care of their older relatives		
My relationship with my older relative was very good prior to his/her old age	0.042	0.182
My current relationship with older relatives is very good and cordial	0.239	<0.001
The nature of my occupation permits me to dedicate enough time to take care of my older relative	0.184	<0.001
I have the financial capacity to take care of my older relative given his/her level of dependency	0.174	<0.001
I have adequate financial resources to cater for the medical needs of my older relative	0.11	<0.001
Am strong enough psychologically to take care of my older relative	0.231	<0.001
I have enough free time that allows me to take care of my older relative without major assistance from other people	0.212	<0.001
I understand my older relative very well, so it is easy for me to take care of him/her	0.277	<0.001
My older relative appreciates every help or support that I offer him/her	0.19	<0.001
I have the capacity to take care of my older given his/her mental/psychological state	0.227	<0.001
My older relative suffers from psychological problems that require my regular presence	0.137	<0.001

Next, the older adults' physical weakness and serious health problems showed a weak positive correlation with the outcome “willingness to consider elderly daycare for your older relative.” Lastly, in Table 4, the correlation between the perceived self-efficacy of caregivers and the degree of willingness to consider elderly daycare facilities was analysed using Kendall’s Tau. The results showed a weak positive correlation with the outcome “willingness to consider elderly daycare for your older relative.”

Ordinal logistic regression

Ordinal logistic regression was done to properly assess which independent variables have a statistically significant impact on the degree of willingness to enrol older relatives in elderly daycare institutions (dependent variable). 

Age was the only factor that showed statistical significance in the multivariable analysis. As compared to participants in the age group 18-26 years, those in the age group 45-51 years showed a higher willingness to consider elderly daycare (OR: 3.85, 95% CI: 1.72 to 8.33) (Table 5).

**Table 5:** Effect of sociodemographic characteristics on the willingness to consider elderly daycare centers for the elderly.

	Univariate				Multivariable			
	OR	P-value	95.0% CI		OR	P-value	95.0% CI	
			Lower bound	Upper bound			Lower bound	Upper bound
Sex								
Male	1.00				1.00			
Female	1.06	0.675	0.81	1.39	0.87	0.385	0.64	1.19
Social situation								
Single	1.00				1.00			
Married	1.27	0.143	0.92	1.75	0.82	0.49	0.47	1.43
Divorced	0.87	0.679	0.45	1.69	0.53	0.113	0.24	1.16
Widowed	1.35	0.294	0.78	2.33	0.93	0.861	0.44	2.00
Age								
18-26 years	1.00				1.00			
27-32 years	1.41	0.115	0.92	2.17	1.75	0.076	0.94	3.23
33-38 years	1.30	0.168	0.89	1.92	1.54	0.174	0.83	2.86
39-44 years	2.13	0.002	1.33	3.45	2.08	0.034	1.05	4.17
45-51 years	3.70	<0.001	2.00	6.67	3.85	0.001	1.72	8.33
52-59 years	1.45	0.12	0.91	2.27	1.43	0.286	0.74	2.78
Source of income								
Formal employment	1.00				1.00			
Casual employment	0.60	0.003	0.42	0.84	0.72	0.103	0.49	1.06
Business	0.75	0.301	0.43	1.30	0.91	0.757	0.49	1.67
Others	0.68	0.048	0.46	1.00	1.05	0.844	0.65	1.69
Monthly income range								
Below 5000	1.00				1.00			
5000-10,000	1.06	0.765	0.72	1.54	1.08	0.755	0.67	1.75
11000-15,000	1.41	0.092	0.94	2.08	1.45	0.172	0.85	2.50
16000-20000	2.04	0.002	1.28	3.23	1.61	0.113	0.89	2.94
21000 and above	1.14	0.662	0.63	2.08	0.93	0.824	0.47	1.85
The size of your household								
From 2-5	1.00				1.00			
From 6-9	0.88	0.402	0.66	1.18	0.98	0.916	0.72	1.35
From 10-12	1.02	0.927	0.69	1.49	1.09	0.689	0.71	1.69
More than 12	0.87	0.81	0.28	2.70	0.63	0.423	0.20	1.96
Education								
Less than university degree	1.00				1.00			
College degree	1.02	0.905	0.71	1.47	0.90	0.601	0.61	1.33
Post-graduate studies	1.69	<0.001	1.27	2.27	1.37	0.071	0.97	1.96

In Table 6, ordinal logistic regression was used to identify the association between public perception of the elderly and willingness to consider elderly daycare. Significantly higher willingness to consider daycare was associated with the public perception that the elderly play an important role in society; they are wise, aware of daycare and accommodation facilities in daycare, financially capable of taking care of and have adequate financial resources to cater for the medical needs of an older relative.

**Table 6:** Public perception of the elderly is associated with their willingness to consider elderly daycare.

	Univariate					Multivariable		
	OR	P-value	95.0% CI		OR	P-value	95.0% CI	
			Lower bound	Upper bound			Lower bound	Upper bound
Public perception of older people								
Older people play important roles in our society	2.78	<0.001	2.33	3.33	1.67	<0.001	1.30	2.13
Older people are wise and knowledgeable about the traditions of their society	2.56	<0.001	2.17	3.03	1.49	0.001	1.19	1.89
Older people are less tolerated and received to changes in society	1.59	<0.001	1.41	1.82	1.14	0.089	0.98	1.33
Older people are being neglected by their dependents in many families	1.32	<0.001	1.16	1.47	1.02	0.796	0.85	1.22
Older people are leading solitary lives in many families	1.33	<0.001	1.18	1.49	1.01	0.868	0.85	1.22
There are many cases of elder abuse in society	1.30	<0.001	1.15	1.45	1.04	0.594	0.89	1.22
Older people are respected and valued in society	1.69	<0.001	1.49	1.92	1.14	0.136	0.96	1.35
Older people are warm and welcoming to the younger generation	1.67	<0.001	1.45	1.92	1.19	0.052	1.00	1.41
Perceived institutional efficacy on care provision for older people								
I am very much aware of the existence of elderly daycare institutions in Saudi Arabia	1.35	<0.001	1.18	1.54	1.37	0.003	1.11	1.67
Elderly daycare institutions have good accommodation facilities to take care of my older relatives	1.16	0.02	1.02	1.32	0.78	0.019	0.63	0.96
Elderly daycare institutions have enough facilities that my older relative can use for physical exercises	1.22	0.001	1.09	1.37	1.09	0.355	0.91	1.32
Elderly daycare institutions for older people are operating according to government regulations	1.28	<0.001	1.12	1.45	1.14	0.176	0.94	1.39
Elderly daycare institutions have sufficient and acceptable programs and activities that my older relative can use for social bonding	1.20	0.002	1.08	1.37	0.93	0.437	0.76	1.12
I have confidence in the individuals/organizations that have established elderly daycare institutions for older people	1.32	<0.001	1.16	1.49	1.18	0.113	0.96	1.43
Elderly daycare institutions have well-trained personnel that can take care of my older relatives	1.28	<0.001	1.12	1.45	0.94	0.568	0.76	1.16
Staff of elderly daycare institutions have positive attitudes toward older people in society	1.35	<0.001	1.19	1.56	1.23	0.055	1.00	1.52
Elderly daycare institutions have adequate staff to respond to the needs of my older	1.22	0.002	1.08	1.39	1.03	0.76	0.86	1.22
Perceived efficacy of members of the public (caregivers) to take care of their older relatives								
The nature of my occupation permits me to dedicate enough time to take care of my older relative	1.43	<0.001	1.27	1.61	1.15	0.039	1.01	1.32
I have the financial capacity to take care of my older relative given his/her level of dependency	1.39	<0.001	1.25	1.56	1.23	0.014	1.04	1.45
I have adequate financial resources to cater for the medical needs of my older relative	1.27	<0.001	1.12	1.43	0.83	0.035	0.69	0.99
Am strong enough psychologically to take care of my older relative	1.59	<0.001	1.39	1.79	1.25	0.008	1.06	1.47
I have enough free time that allows me to take care of my older relative without major assistance from other people	1.54	<0.001	1.37	1.75	1.14	0.096	0.98	1.33
I understand my older relative very well, so it is easy for me to take care of him/her	1.79	<0.001	1.56	2.04	1.37	0.001	1.14	1.67
My older relative appreciates every help or support that I offer him/her	1.47	<0.001	1.30	1.67	1.01	0.954	0.85	1.19
I have the capacity to take care of my older given his/her mental/psychological state	1.64	<0.001	1.45	1.89	1.18	0.063	0.99	1.41
My older relative suffers from psychological problems that require my regular presence	1.35	<0.001	1.19	1.54	1.10	0.183	0.95	1.28

## Discussion

Daycare for older adults is a relatively new concept in Saudi Arabia; however, the Saudi population is carefully considering it for their elderly relatives. The study found that about 67% are willing to enrol their older family members in daycare facilities. Yet, it is important to note that there are individuals who may have concerns about daycare centres due to a lack of knowledge or the belief that a family setting can offer superior care compared to an institutional environment. Conversely, these individuals may also be apprehensive about the potential challenges and adjustments that come with transitioning their loved ones into a daycare facility. In comparison to other countries, Saudi Arabia has a much higher willingness to embrace elderly daycare; for instance, in a Kenyan study conducted in Nakuru County, only 17% of respondents were willing to take their older relatives to daycare facilities [12]. However, in Kenya and most other African countries, the population is young, and they are less aware of ageing issues in society. Chepkwony agrees that younger people in a communal setting are obligated to look after the elderly, and parents ought to remain with their families in these kinds of communities. In contrast, sending them to daycare suggests they either avoid their responsibility or want to get rid of older members [12].

Since the Saudi community is based on the extended family, who form the primary caregivers to older relatives, sending them to daycare may not be a common practice. Therefore, the idea of sending older relatives to daycare centres may be seen as neglect or abandonment, creating a perception of shame among the elderly. However, willingness to consider daycare for older relatives in Saudi Arabia does not appear to vary with gender, education, income, or social status, but evidence from this study confirms that the willingness to consider elderly daycare for an older relative is influenced significantly by age. Persons who are between 39 to 51 years old are usually working full-time jobs, which reduces the amount of care they can provide to an older relative due to the limited time and energy. According to Scharlach et al. (2007), as employed caregivers cannot provide a large amount of care, they are more likely to consider care supplementation either through a formal care provider such as adult daycare or by asking family and friends for assistance [13].

Elderly care in Saudi Arabia faces cultural challenges due to strong societal norms and the important role families take towards their elders. Elderly daycare centres are often stigmatised and are perceived as conflicting with religious and traditional norms in the context, which emphasise family-based caregiving. As a result, sometimes elderly care centres are viewed as a neglect of familial duties, leading to societal judgement and limited acceptance [14]. Elderly daycare centres can significantly improve the quality of life for older adults by enhancing their social well-being, encouraging interaction, and engaging elders in activities. These centres also support family caregivers by giving them relief from the full family responsibility [15]. For a better outcome, elderly daycare centres in Saudi Arabia must adapt cultural values, incorporate familiar elements, and show that they complement, rather than replace, family care. Raising community awareness and providing government support, such as financial assistance and transportation, can also help make these centres more accessible and accepted [10,16].

The other main reason Saudis are willing to send their elderly relatives to daycare is their high social status. Most citizens believe that older people deserve the best care because they play an important role in society. In the traditional Saudi community, more senior members held the community together and were the source of collective knowledge passed down from generation to generation. Similarly, most citizens agreed that the elderly are wise and knowledgeable about Saudi traditions and culture. While it is true that some individuals may argue that older adults have less value in society due to societal shifts towards individualistic lifestyles and a focus on education, it is important to recognise that older adults still possess valuable knowledge and wisdom that cannot be solely acquired through formal education. Their life experiences and collective knowledge are essential for maintaining cultural traditions and societal cohesion. Furthermore, older adults continue to contribute to society by serving as mentors, advisors, and leaders in their communities [17].

Furthermore, 62% of the participants agreed that they understand their older relatives very well, which makes it easy for them to provide the needed care. In addition, half of the participants agreed that they don’t have the financial resources to cater for the medical needs of the elderly and cannot meet the cost of their care since they suffer from serious health problems that require continuous and intensive medical care. The high price could increase the cost of daycare or make them unable to care for such patients [18]. Thus, most families will prefer hospitals over daycare to take their older relatives suffering from life-long illnesses such as heart failure and systemic lupus [19,20]. 

Conversely, the increased receptiveness in Saudi Arabia regarding daycare comes from an increased consciousness of the mental and physical health of the elderly. The Kingdom’s vision of the 2030 health sector transformation program “aims to restructure the health sector in Saudi Arabia to be a comprehensive, effective, and integrated health system that is based on the health of the individual and society” [21]. In the last few years, Saudi Arabia has been focusing on elderly mental and physical health needs; therefore, elderly daycare centres have emerged as one of the newest healthcare providers that will minimise the pressure off the current healthcare system [22]. The concept of daycare is recent in Saudi Arabia, which explains the hesitation in society to accept them. More than three-quarters of the population believe that although young family members are receptive to the idea of daycare and are willing to take their older relatives to them, some still prefer traditional family support or seek professional medical help in hospitals when needed [19] as they believe that elderly can receive attention and quality care in the hospital compared to daycare facilities [20].

Daycare can positively impact the mental and physical health of older adults [20]. Understanding and respecting the cultural and religious practices of Saudi Arabia is essential in providing effective home care that aligns with daycare services. By incorporating these considerations into the care provided, it ensures a more inclusive and culturally sensitive environment for all individuals involved [10]. Although most Saudis do not believe they neglect their older relatives, more than half indicate that the elderly live an increasingly solitary life, which can have detrimental effects on their mental health. A study conducted by Dukhaykh and Bilimoria showed that as more Saudis spend most of their time outdoors for employment and education pursuits, older relatives remain at home alone, contributing to the perception of loneliness [23]. However, Al-Abadi claims that although daycare can result in social isolation of older adults because they lose relationship contact and regular communication with family members, it can also lead to increased communication with peers, which can benefit their mental health [24]. Additionally, daycare facilities provide senior citizens with convenient access to medical care and counselling, which can improve their psychological and physical health and well-being [25]. Finally, good accommodations that can accommodate the elderly's daily needs and lower their risk of falling (a condition that can seriously harm their health) must be provided by daycare facilities. Thus, daycare facilities may lessen the amount of social contact between the elderly and their families; they provide them with opportunities to socialise with their peers and receive appropriate healthcare, an accident-free environment, and programs and activities to keep them fit, contributing to a better quality of life [26].

Strengths and limitations of the study

To the best of our knowledge, this is one of the few research studies discussing elderly daycare in Saudi Arabia. The information included in this study can be used as a reference for decision-makers in the healthcare field to improve the services provided to the elderly. Some limitations included a lack of previous research studies on the topic and the difficulty of contacting the study sample to answer the study questionnaire. In addition, cross-sectional design only allows for a snapshot of data at one point in time, limiting the ability to establish causality or track changes over time. Additionally, cross-sectional studies may not capture the full complexity of variables and relationships that could be better understood through longitudinal research. It is important to acknowledge that not all individuals have access to or are active on social media, which may result in a skewed sample. Additionally, the lack of control over who sees the recruitment posts can lead to an unrepresentative sample. Further, we didn’t collect information about how many elderly people were there in the whole household size.

## Conclusions

Daycare facilities for the elderly are gradually emerging in the Saudi community. While much older people are reluctant to seek their services, a growing number of younger people believe daycare centres have the expertise and resources to take care of their older relatives. Conversely, about a quarter of the Saudi population is unwilling to send their older relatives to daycare facilities because they lack knowledge about them and how they work. Thus, one approach the government can take to improve daycare use is educating the public about its importance in Saudi society. Accurate information could also gradually lead to a reduced sense of shame associated with sending a family member to daycare for the elderly. Hence, education is the first strategy the Saudi government can take to inform the public about the importance of daycare for the elderly and encourage greater uptake of this service.

The second approach is to find out the needs of the elderly and design daycare facilities that meet all their needs. As indicated, a large number of Saudis would rather care for their elderly in hospitals than daycare centres. They believe that hospitals have the expertise and resources to manage all health conditions that might be beyond the services provided by daycare facilities. Such a belief could lead to straining hospital resources and denying care to patients who require medical supervision. A potential strategy for the Saudi government to enhance the utilisation of daycare services is conducting research on the daily requirements of senior citizens and setting up daycare facilities equipped with all the amenities, personnel, and equipment needed to address their social, medical, and physical needs. In so doing, the government can encourage Saudis to take their older relatives to daycare facilities over the more costly hospitals, which lack peer socialisation. Another possible approach can be home daycare services, like those in the UK, being introduced in Saudi Arabia to provide more personalised care for the elderly in the comfort of their own homes. This model may offer a convenient and flexible option for families looking for alternative caregiving solutions.

## Appendices

**Table 7:** Study questionnaire about assessment of the population's attitude toward elderly daycare centres in Saudi Arabia.

PART A: Demographic data	
State your gender	Male Female
State your marital status	Single Married Widowed Divorced
State your age bracket	18-26 Years 27-32 Years 33-38 Years 39-44 Years 45-51 Years 52-59 Years
State your main source of income	Formal employment Casual employment Business Others
State your cumulative monthly income range	Below 5000 5000-10000 11000-15,000 16000-20000 21000-25000 26000-30000 31000 and above
What is the size of your household	2-5 6-9 10-12 13-15 More than 15
State your highest level of formal education	No formal education Primary Secondary High School College University Masters or PhD
To what extent are you willing to consider elderly daycare for your older relative?	Very willing Willing Somehow willing Least willing Not willing

	Strongly agree	Agree	Somehow agree	Disagree	Strongly disagree
Part B. Public perception of older people					
Older people play important roles in our society					
Older people are wise and knowledgeable about the traditions of their society					
Older people are less tolerated and received to changes in society					
Older people are being neglected by their dependents in many families					
Older people are leading solitary lives in many families					
There are many cases of elder abuse in society					
Older people are respected and valued in society					
Older people are warm and welcoming to the younger generation					
Part C. Perceived institutional efficacy on care provision for older people					
I am very much aware of the existence of elderly daycare institutions in Saudi Arabia					
Elderly daycare institutions have good accommodation facilities to take care of my older relatives					
Elderly daycare institutions have enough facilities that my older relative can use for physical exercises					
Elderly daycare institutions for older people are operating according to government regulations					
Elderly daycare institutions have sufficient and acceptable programs and activities that my older relative can use for social bonding					
I have confidence in the individuals/organizations that have established elderly daycare institutions for older people					
Elderly daycare institutions have well-trained personnel that can take care of my older relatives					
Staff of elderly daycare institutions have positive attitudes toward older people in society					
Elderly daycare institutions have adequate staff to respond to the needs of my older					
Part D. Perceived older people’s efficacy on care provision for themselves					
My older relative is physically weak and cannot fend for him/herself					
My older relative suffers from serious health problems that require regular medical checkups					
My older relative is economically unstable and cannot meet the cost of his/her medical needs (financial capacity)					
My older relative does not have enough friends to give him/her company (psychosocial status)					
Part E. Perceived efficacy of members of the public (caregivers) to take care of their older relatives					
The nature of my occupation permits me to dedicate enough time to take care of my older relative					
I have the financial capacity to take care of my older relative given his/her level of dependency					
I have adequate financial resources to cater for the medical needs of my older relative					
Am strong enough psychologically to take care of my older relative					
I have enough free time that allows me to take care of my older relative without major assistance from other people					
I understand my older relative very well, so it is easy for me to take care of him/her					
My older relative appreciates every help or support that I offer him/her					
I have the capacity to take care of my older given his/her mental/psychological state					
My older relative suffers from psychological problems that require my regular presence					
